# Laparoscopic resection of a metachronous secondary lymph node metastasis in the mesentery of the ileum after surgery for sigmoid colon cancer with ileum invasion: a case report

**DOI:** 10.1186/s40792-021-01114-w

**Published:** 2021-01-25

**Authors:** Seiichiro Eto, Nobuo Omura, Tetsuya Shimada, Teruyuki Takishima, Hideyuki Takeuchi, Wataru Kai, Keita Kodera, Tomo Matsumoto, Tsuyoshi Hirabayashi, Hidejiro Kawahara

**Affiliations:** 1 Department of Surgery, National Hospital Organization Nishisaitama-Chuo National Hospital, Wakasa 2-1671, Tokorozawa-shi, Tokyo, Saitama 359-1151 Japan; 2 Department of Surgery, The Jikei University School of Medicine, Tokyo, Japan; 3 Department of Pathology, National Hospital Organization Nishisaitama-Chuo National Hospital, Tokyo, Japan

**Keywords:** Locally invasive colon cancer, Metachronous secondary lymph node metastasis, Small mesentery lymph node metastasis

## Abstract

**Background:**

Extended excision of the permeation organ neighborhood is often performed in locally invasive colon cancer, and it is reported to have a survival benefit. In addition, some cases of secondary lymph node metastases in a permeation organ were reported. However, they are reports of synchronous secondary lymph node metastases, not metachronous secondary lymph node metastases. To the best of our knowledge, there are no cases of metachronous secondary lymph node metastases after the resection of a primary colorectal cancer in PubMed.

**Case presentation:**

The case was a 67-year-old man who underwent colonoscopy because of weight loss. Sigmoid colon cancer with all circumference-related stenosis was found by examination, and the patient was transferred to our hospital for the purpose of scrutiny and treatment. The small intestine ileus caused by the invasion of sigmoid colon cancer developed after the transfer. Laparoscopic high anterior resection and extended excision of small intestine segmental resection was performed after the intestinal tract decompression with a nasal ileus tube. Histopathological analysis revealed a pathological diagnosis of pT4b (ileal submucosal invasion) N0 (0/11) M0 f Stage II, tub2, ly1, v2, PN0. Although adjuvant chemotherapy with capecitabine after the operation was planned for half a year, treatment was suspended in the first course by the patient’s self-judgment. No recurrence was observed for a year after the operation, but metastasis recurrence in the para-aortic lymph node was found by a computed tomography (CT) one and a half years after the operation. 18 F-fluorodeoxyglucose (FDG) positron emission tomography revealed that FDG was accumulated only in the para-aortic lymph node. Laparoscopic metastasis lymphadenectomy was performed due to the diagnosis of metachronous metastasis to the para-aortic lymph node alone. Intraoperative findings revealed that lymph node metastasis occurred in the mesentery of the ileum. No adjuvant treatment was done after the secondary operation, and he is still alive with no recurrence 1 year and 9 months after the operation.

**Conclusions:**

We report a rare case of a laparoscopic resection of a metachronous secondary lymph node metastasis in the mesentery of the ileum after surgery for sigmoid colon cancer with ileum invasion.

## Background

Extended excision of the permeation organ neighborhood is often performed in locally invasive colon cancer, and it is reported to have a survival benefit [1, 2]. In addition, some cases of secondary lymph node metastases in a permeation organ were reported [3–7]. However, they are the reports of synchronous secondary lymph node metastases, not metachronous secondary lymph node metastases. There were no published cases of metachronous secondary lymph node metastases after the resection of a primary colorectal cancer when we searched in PubMed.

## Case presentation

**Fig. 1:**
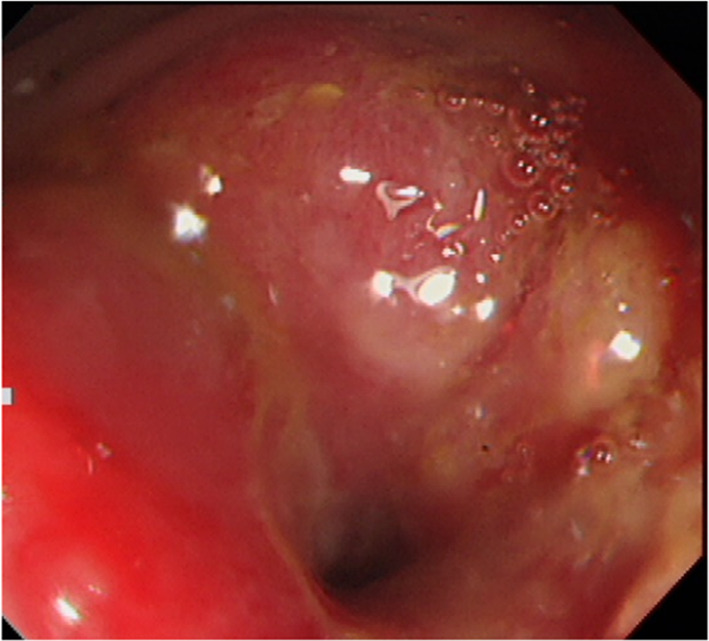
Colonoscopy revealed a sigmoid colon cancer with all circumference-related stenosis

**Fig. 2:**
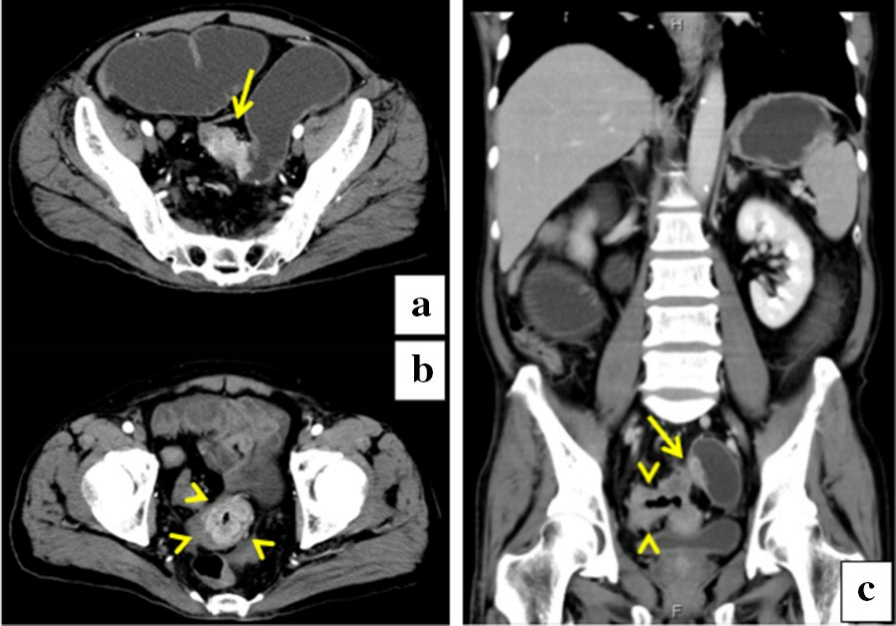
**a–c** CT revealed a 6.5-cm-sized sigmoid colon cancer. Tumor invasion was extended to the ileum and caused the small intestine ileus. The arrowhead shows sigmoidal tumor, and the arrow shows ileal invasion

**Fig. 3:**
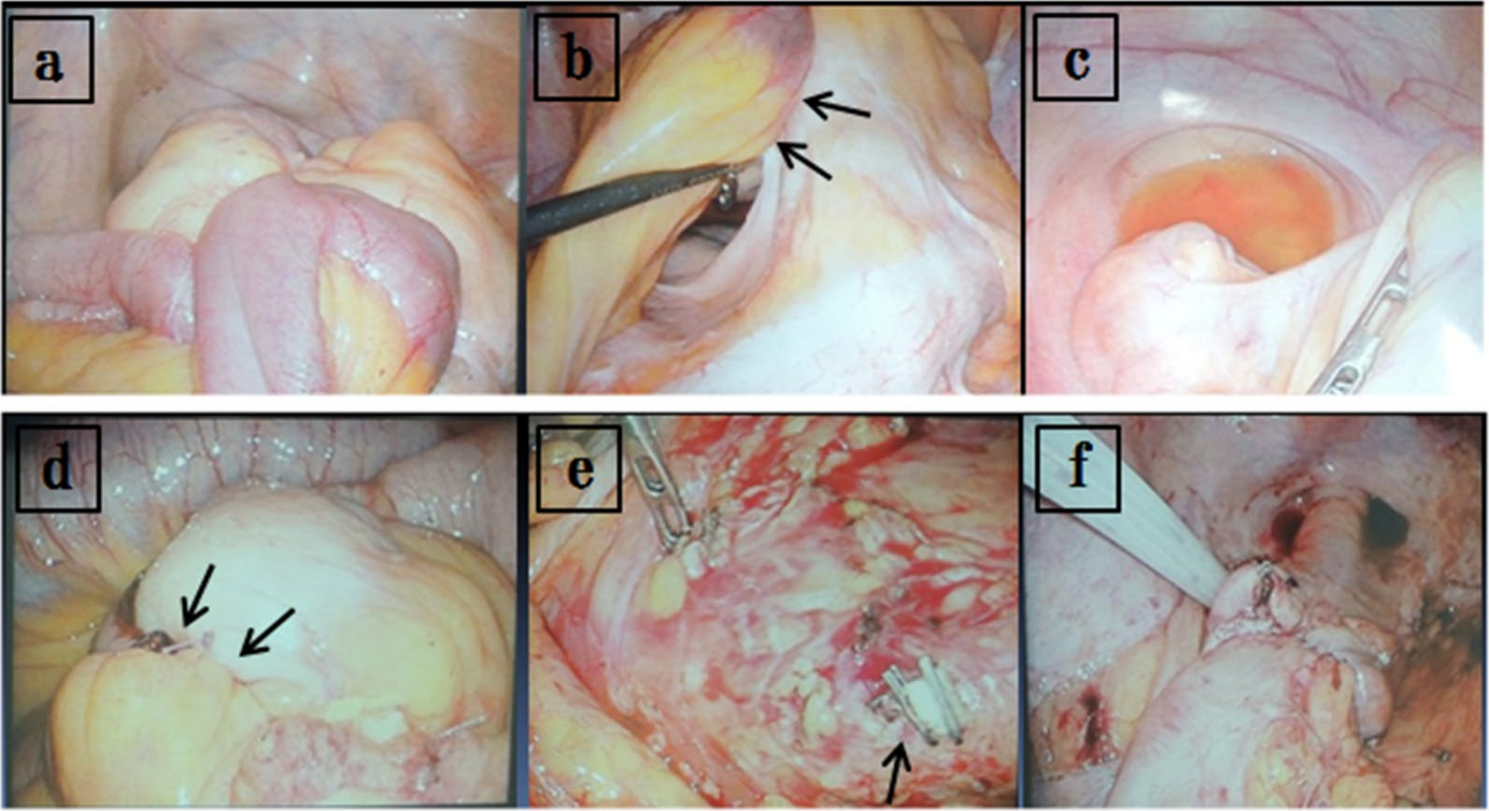
**a, b** Tumor invasion was extended to the ileum. The arrow shows ileal invasion caused by sigmoidal tumor. **c** A small amount of ascites existed in the pouch of Douglas. **d** The invaded ileum was resected with sigmoid colon cancer. Ileal mesentery resection was performed to a minimum, and the arrow shows ileal mesenteric suture. **e, f** Laparoscopic high anterior resection with D3 lymph node dissection was performed. The arrow shows inferior mesenteric arterial root

**Fig. 4:**
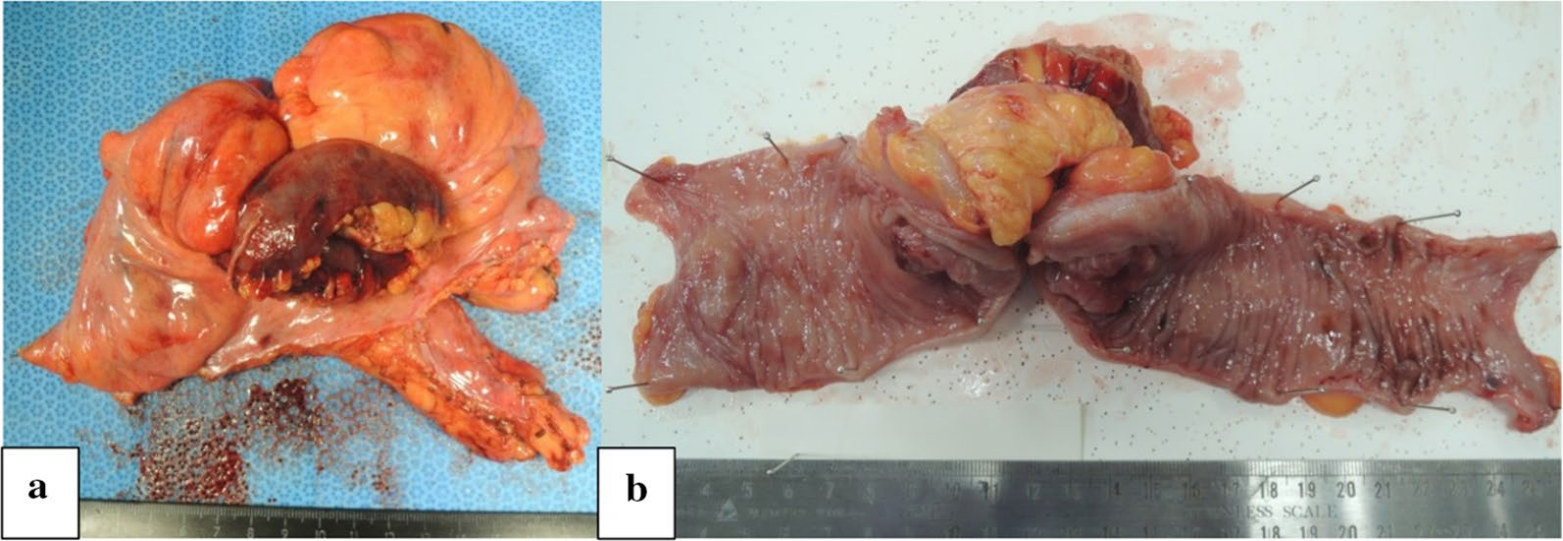
**a, b** Sigmoid colon cancer with invaded ileum was resected en bloc. Ileal mesentery resection was performed to a minimum

**Fig. 5:**
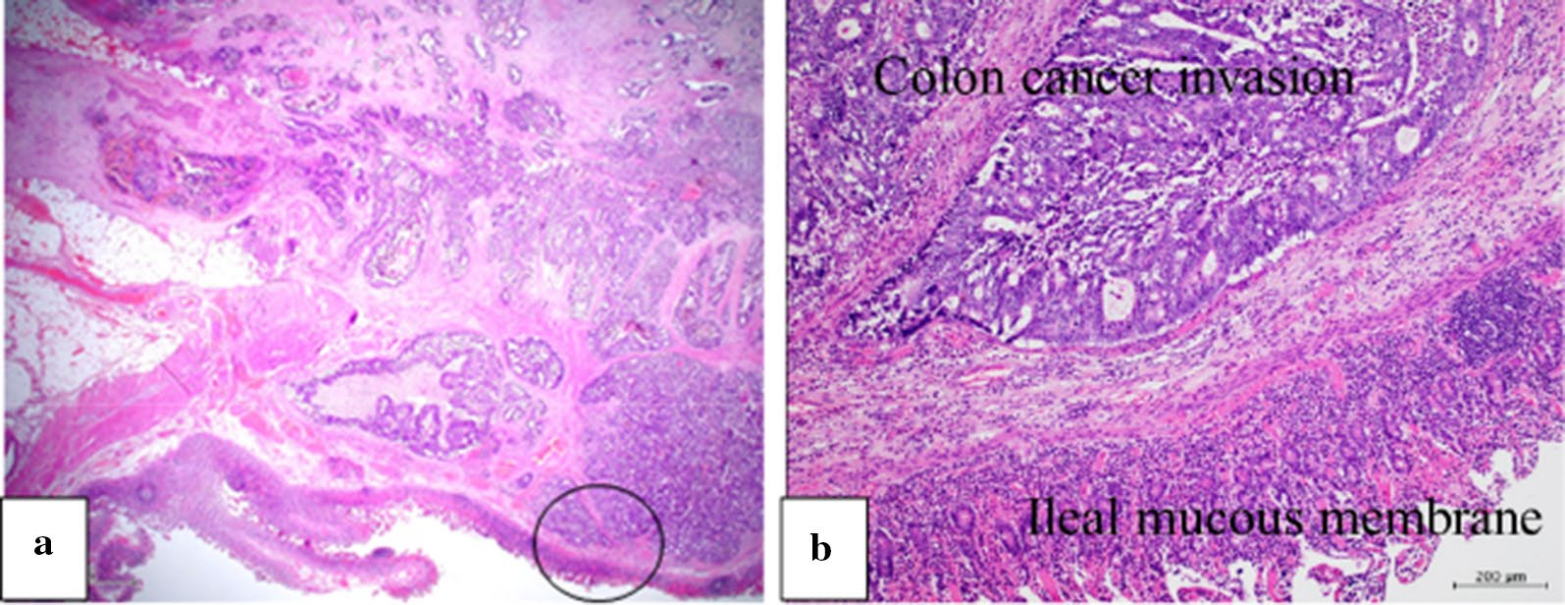
**a, b** Histopathological analysis revealed that tumor invasion was extended to the ileal submucosa. **a** H&E, × 1.25, **b** H&E, × 40

**Fig. 6:**
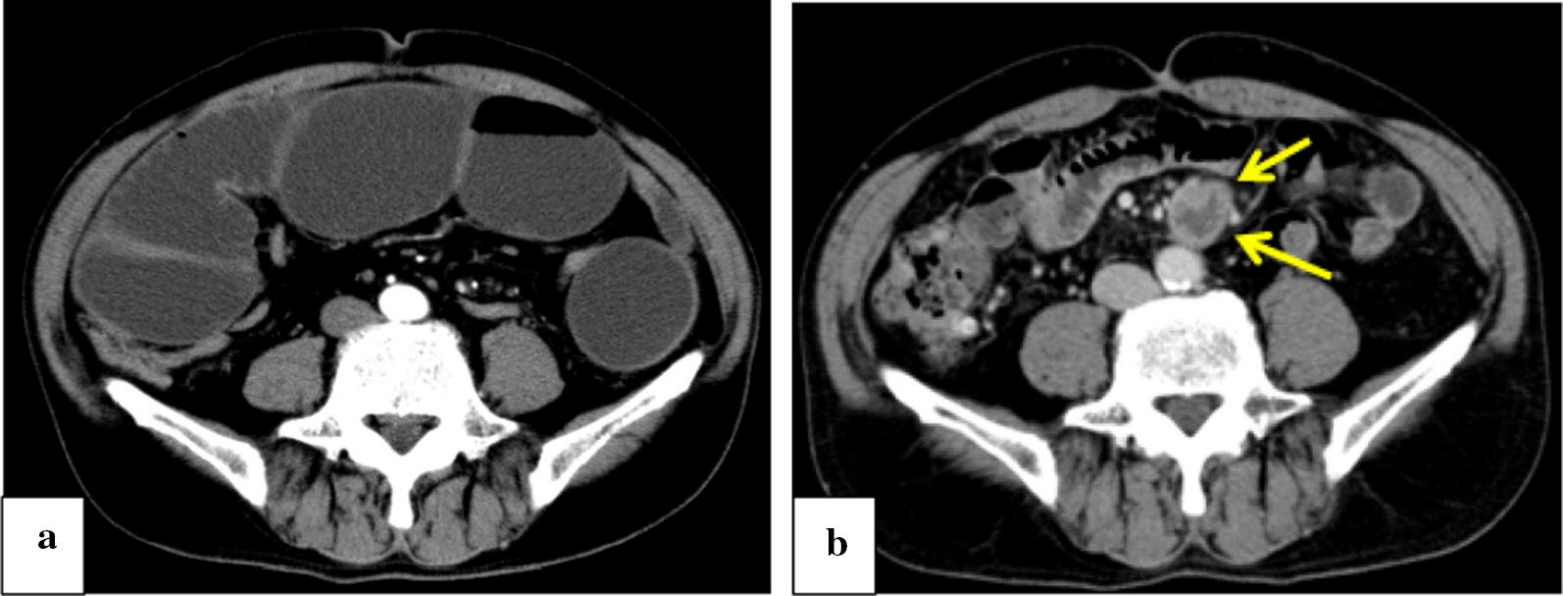
**a** Metastasis in the para-aortic lymph node was not observed by the preoperative CT scan. **b** The para-aortic lymph node metastasis was found by a follow-up abdominal contrasting CT scan one and a half years after the operation. The arrow shows the lymph node metastasis

**Fig. 7:**
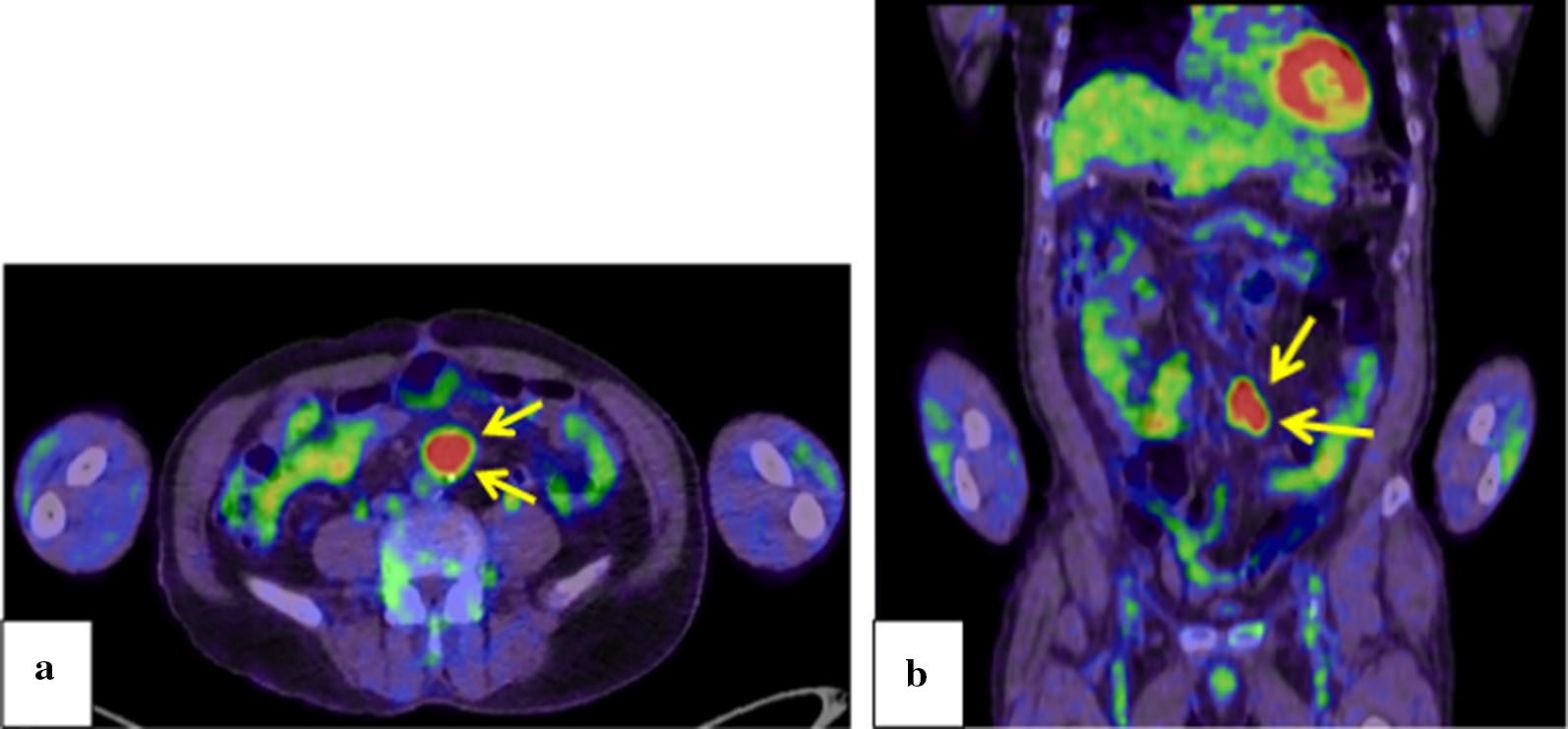
**a, b** PET revealed FDG accumulated only into the para-aortic lymph node

**Fig. 8:**
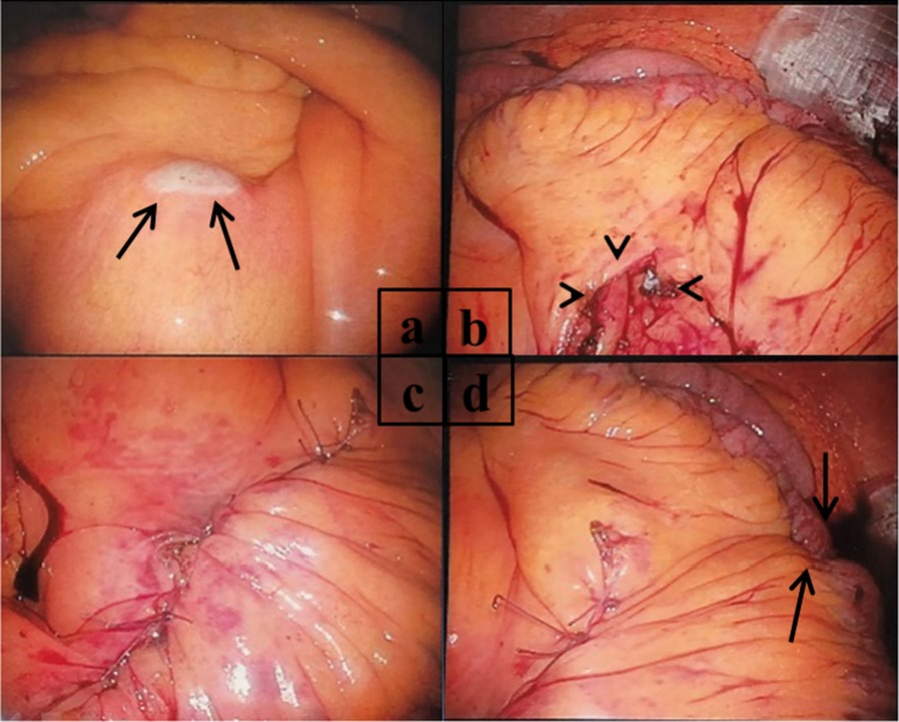
**a** Intraoperative findings revealed that lymph node metastasis occurred in the mesentery of the ileum. The arrow shows the lymph node metastasis in the ileal mesentery. **b–d** Lymphadenectomy was performed, and the arrowhead shows the defect of ileal mesentery after lymphadenectomy. The arrow shows the anastomosis where the first operation of the partial resection of invaded ileum was done, and it was near the lymph node metastasis location

**Fig. 9:**
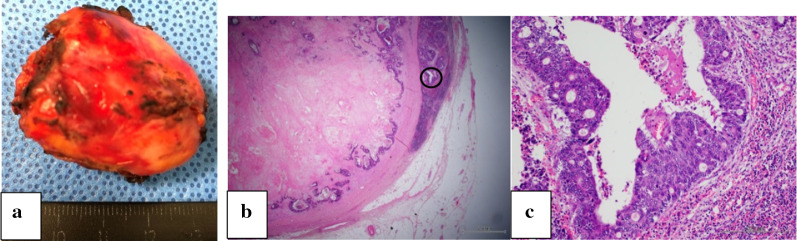
**a** Resected specimen. **b, c** Histopathological analysis was consistent with a metastatic lymph node by colon cancer recurrence as moderately differentiated tubular adenocarcinoma. **b** H&E, × 1.25, **c** H&E, × 10

A 67-year-old man underwent colonoscopy because of weight loss and nausea. Sigmoid colon cancer with all circumference-related stenosis was found by the examination (Fig. 1), and the patient was transferred to our hospital for an additional close inspection and medical treatment. There was no medical history for him. None of his family had a clear history of cancer. Hematological examination showed no elevation of inflammation. The serum carcinoembryonic antigen (CEA) was 5.5 μg/ml (normal range < 5.0 μg/ml) and the serum carbohydrate antigen 19-9 (CA19-9) was 7.8 U/ml (normal range < 37 U/ml). The small intestine ileus caused by the invasion of sigmoid colon cancer to the ileum developed after the transfer (Fig. 2a–c). The maximum tumor diameter was 6.5 cm, sized by computed tomography (CT) scan (Fig. 2c). The decompression of the small intestine was performed before the surgery using a nasal ileus tube. Laparoscopic high anterior resection and extended excision of small intestine segmental resection were performed (Fig. 3a, b, d, e, f). A small amount of ascites existed in the pouch of Douglas (Fig. 3c), and the operative rapid pathologic diagnosis examination showed class I. We firstly divided the small intestine at the point of oral and anal 10 cm distance from the invaded region. Secondly, we dissected the ileal mesentery using an energy device. Thirdly, we performed usual inner approach of high anterior resection for the sigmoid colon cancer. Protective laparoscopic operation was done, and the sigmoid colon cancer with invaded ileum was resected en bloc. Because there was no sign of lymph node metastasis in the ileal mesentery, ileal mesentery resection was performed to a minimum (Fig. 4a, b). The operation time was 294 min, and the blood loss volume was 32 ml. Histopathological analysis revealed a pathological diagnosis as pT4b (ileal submucosal invasion) N0 (0/11) M0 f Stage II, moderately differentiated tubular adenocarcinoma, ly1, v2, PN0 (Fig. 5a, b). The postoperative course was good, and he was discharged on the 13th postoperative day. Although adjuvant chemotherapy with capecitabine after the operation was planned for half a year, treatment was suspended in the first course due to the patient’s self-judgment. We enforced contrasting CT scan every 6 months. No recurrence was observed for a year after the operation, but metastasis recurrence in the para-aortic lymph node was found by a follow-up abdominal contrasting CT scan one and a half years after the operation (Fig. 6b). Metastasis in the para-aortic lymph node was not observed by preoperative CT scan (Fig. 6a). The tumor markers at that time were at normal levels (CEA was 3.4 μg/ml and CA19-9 was 2.6 U/ml). ^18^F-fluorodeoxyglucose (FDG) positron emission tomography (PET) revealed that FDG accumulated only in the para-aortic lymph node (Fig. 7a, b). Although there was the possibility of dissemination of sigmoidal cancer, we decided to perform surgical resection because the tumor marker level was normal and FDG was not accumulated in any other place. Because there were no findings about small intestine carcinoma in PET–CT and no clinical symptoms of small intestine carcinoma like progression of anemia and elevated tumor markers, we did not review the small intestine for carcinoma by the enteroscopy. Laparoscopic metastasis lymphadenectomy was performed due to the diagnosis of metachronous metastasis to the para-aortic lymph node alone. Intraoperative findings revealed that lymph node metastasis occurred in the mesentery of the ileum where we had performed the partial resection of invaded ileum in the first operation. The lymph node metastasis was located central side of the anastomotic intestine mesentery and near the marginal artery. Protective laparoscopic operation was done and we could preserve the marginal artery (Fig. 8a–d). The operation time was 160 min, and the blood loss volume was low. Histopathological analysis was consistent with a lymph node metastasized by colon cancer recurrence as moderately differentiated tubular adenocarcinoma (Fig. 9a, b). The postoperative course was good, and he was discharged on the 8th postoperative day. Although we recommended adjuvant chemotherapy after the second surgery, the patient refused. No adjuvant treatment was given after the secondary operation, and he is still alive with no recurrence 1 year and 9 months after the first operation.

## Discussion

There are few clear definitions of “metachronous secondary lymph node metastasis” in previous case reports, so here, we define “metachronous” and “secondary lymph node metastasis”. “Metachronous” indicates that the cancers follow in sequence, that is, more than 6 months apart. “Secondary lymph node metastasis” is lymph node metastasis that occurs in adjacent organs’ regional lymph nodes. Senda and others accumulated 27 reported cases in Japan and examined the characteristics of invasive colon cancer [8]. Characteristics of the invasive colon cancer were “large tumor diameter”, “few lymph node metastases although the most of them were advanced colon cancer” and “rarity of liver metastasis”. In addition, Ueno and others retrospectively analyzed 90 cases of invasive colorectal cancer in their own facility [9]. The rate of all circumference-related stenosis cases was significantly high (82.2%) in invasive colorectal cancer patients. The rate of cases in which the maximum tumor diameter was over 5.1 cm was significantly high (85.6%) in invasive colorectal cancer patients. Mucinous carcinoma and poorly differentiated adenocarcinoma are more likely to reflect extramural growth than well-differentiated adenocarcinoma, regarding histopathological findings [10–12]. However, there are not enough reports about the mechanism of secondary lymph node metastasis in locally invasive colon cancer [13]. Ueno and others analyzed clinical cases of invasive colon cancer with synchronous secondary small mesentery lymph node metastases [9]. They determined that synchronous secondary small mesentery lymph node metastases occurred when the tumor invasion involved the small intestine mucous membrane and when the fistula was formed. There was a case in which synchronous secondary small mesentery lymph node metastases were detected without metastasis in colon cancer regional lymph nodes. They also reported the prognosis of patients with synchronous secondary lymph node metastasis. Although one case died of hematogenous metastasis in 1 year and 3 months, two cases had a long-term prognosis without recurrence after radical resection of the tumor (Table 1). It is difficult to presume the prognosis of our case, but we think that radical resection of metachronous secondary lymph node metastasis leads to good prognosis. Current literature does not provide a clear answer on the oncological safety of laparoscopic surgery for locally advanced colon cancer. Charlotte and others reported a systematic review and meta-analysis of laparoscopic surgery for T4 colon cancer [14]. Although they reported that no significant differences in any survival measures were found between laparoscopic and open surgery for T4 colon cancer, they also concluded that laparoscopic surgery for T4b colon cancer seemed less appropriate and it should probably only be performed in selected patients by experienced surgeons. We searched in PubMed with the keywords “locally invasive colon cancer” and “metachronous secondary lymph node metastasis”. There were no cases of metachronous secondary lymph node metastases after the resection of a primary colorectal cancer as far as we searched in PubMed. The characteristics of our case were “invasive sigmoid colon cancer”, “moderately differentiated tubular adenocarcinoma”, “maximum tumor diameter was 6.5 cm (> 5.1 cm)”, “no metastasis in colon cancer regional lymph node”, and “tumor invasion extended to the ileal submucosa”. In the literature, it was thought that metachronous secondary lymph node metastasis in the mesentery of the ileum was caused by ileal microlymphatic vessel invasion. The reason why metachronous and not synchronous metastasis occurred was thought to be because the tumor invasion did not extend to the small intestine mucous membrane. According to previous case reports and our experience of this case, we determined that the characteristics of metachronous secondary lymph node metastases were “invasive colon cancer”, “tumor invasion extended to the small intestine submucosa”, “histology type was not well-differentiated tubular adenocarcinoma”, “maximum tumor diameter was over 5.1 cm” and “few lymph node metastases in colon cancer regional lymph node although the most of them were advanced colon cancer”. When invasion of colon cancer is suspected to reach the submucosa of the small intestine, it is necessary to resect the regional lymph node of the invaded small intestine. We thought that occurrence of the metachronous and not synchronous secondary lymph node metastasis was very rare.

**Table 1:** Cases of secondary lymph node metastasis

Case	References	Year	Sex	Age	Primary location	Differentiation	Invaded organ	Invasion depth of small intestine	Primary regional lymph node metastasis	Recurrence	Outcome (months)
1	Ueno et al. [9]	1990	F	67	Sigmoid	tub1	Ileum	Mucous membrane (forming fistula)	(−)	None	Alive (46)
2	Ueno et al. [9]	1990	F	61	Sigmoid	muc	Ileum	Mucous membrane (forming fistula)	(+)	None	Alive (12)
3	Ueno et al. [9]	1990	M	72	Sigmoid	tub1	Ileum	Mucous membrane	(+)	Lung and liver metastasis	Dead (15)
4	Our case	2020	M	67	Sigmoid	tub2	Ileum	Submucosa (causing obstruction)	(−)	None	Alive (21)

## Conclusions

We report a rare case of a laparoscopic resection of a metachronous lymph node secondary metastasis in the mesentery of the ileum after the surgery for sigmoid colon cancer with ileum invasion.

## Data Availability

Data sharing is not applicable to this article as no datasets were generated or analyzed during the current study.

## Abbreviations

CT: Computed tomography
FDG: F-fluorodeoxyglucose
PET: Positron emission tomography
CEA: Carcinoembryonic antigen
CA19-9: Carbohydrate antigen 19-9
H&E: Hematoxylin and eosin stain
